# Smoothed Temporal Atlases of Age-Gender All-Cause Mortality in South Africa

**DOI:** 10.3390/ijerph14091072

**Published:** 2017-09-15

**Authors:** Samuel O. M. Manda, Nada Abdelatif

**Affiliations:** 1 Biostatistics Unit, South African Medical Research Council, 1 Soutpansberg Road, Pretoria 0001, South Africa; 2 School of Mathematics, Statistics, and Computer Science, University of KwaZulu-Natal, Pietermaritzburg 3209, South Africa; 3 Biostatistics Unit, South African Medical Research Council, Durban 4091, South Africa; nada.abdelatif@mrc.ac.za

**Keywords:** all-cause mortality, spatial-temporal model, Bayesian modelling, South Africa

## Abstract

Most mortality maps in South Africa and most contried of the sub-Saharan region are static, showing aggregated count data over years or at specific years. Lack of space and temporral dynamanics in these maps may adversely impact on their use and application for vigorous public health policy decisions and interventions. This study aims at describing and modeling sub-national distributions of age–gender specific all-cause mortality and their temporal evolutions from 1997 to 2013 in South Africa. Mortality information that included year, age, gender, and municipality administrative division were obtained from Statistics South Africa for the period. Individual mortality level data were grouped by three ages groups (0–14, 15–64, and 65 and over) and gender (male, female) and aggregated at each of the 234 municipalities in the country. The six age-gender all-cause mortality rates may be related due to shared common social deprivation, health and demographic risk factors. We undertake a joint analysis of the spatial-temporal variation of the six age-gender mortality risks. This is done within a shared component spatial model construction where age-gender common and specific spatial and temporal trends are estiamted using a hierarchical Bayesian spatial model. The results show municipal and temporal differentials in mortality risk profiles between age and gender groupings. High rates were seen in 2005, especially for the 15–64 years age group for both males and females. The dynamic geographical and time distributions of subnational age-gender all-cause mortality contribute to a better understanding of the temporal evolvement and geographical variations in the relationship between demographic composition and burden of diseases in South Africa. This provides useful information for effective monitoring and evaluation of public health policies and programmes targeting mortality reduction across time and sub-populations in the country.

## 1. Background

High-quality mortality statistics are needed in optimal health planning, decision-making, program evaluation, progress monitoring, and resource allocation [1,2]. These data are often only reported at high (national) levels of geographic aggregation but not at lower administrative levels for local public health decision making. This is despite substantial evidence pointing to variations in mortality risks at subnational levels due to, for example, age, gender, and social economic differences [3,4]. Additionally, their use both at national and sub-national levels has been limited, as many atlases showing geographic distribution of mortality are stationary even though the data has been collected over many years [5,6,7]. This is despite the recent developments and applications of spatial-temporal methods for disease mapping in spatial epidemiologic studies, arising from the work of Clayton and Kador, Besag, York, and Mollie, and Knorr-Held and Best [8,9,10]. These models have been extended and applied to a variety of disease settings (Richardson et al., Tzala and Best, Feltbower et al., Manda et al. and Ibanez-Beroiz et al. [3,4,11,12,13] to name a few). Additionally, advances in computer systems, the availability of powerful geographical information systems (GIS), and the implementation of many types of spatial models in specialized software have led to a greater uptake of small-area disease mapping.

Most mortality atlases fail to properly utilize the basic elements of disease mapping models to account for both spatial and time dependence in mortality risks. As pointed out in Ocaña-Riola and Mayoral-Cortés [14], several mortality atlases describe the geographical distribution of mortality by grouping year on year data into a single period, even though the data may span over 20 years. Most atlases use age- and gender-adjusted rates or standardized mortality rates, which for small areas or rare causes of death such as cancer, may be unreliable and unstable (Manda et al. and references therein [13]). Moreover, these mortality rate indicators may be invalid when applied to different strata in subnational areas. Burden of diseases and demographic and many factors related to the wellbeing of populations are dynamic and change over time [14]. Thus, the use of static maps of mortality may adversely impact interpretations and decision-making processes regarding health status of the general population and/or specific populations, the effectiveness of programmes, and inequities in health between places and populations [7]. Time effects on mortality should also account for the fact that adjacent periods may have similar rates. This is justified, as mortality rates in adjacent years are more likely to be similar since both mortality counts and population denominators would not have changed considerably. Similarly, mortality rates in adjacent areas could not be assumed to be independent of each other. For rare mortality events and sparse populations, sub-population mortality rates could be very unstable. These issues could be addressed effectively within a spatiotemporal modelling of mortality rates to permit an assessment of the evolution of mortality dependence on both space and time. Moreover, these models help to address the problem of missing and unmeasured ecological determinants of mortality [15,16]. 

In order to provide robust perspectives of mortality over a spectrum of relevant subgroups, disease mapping models for the rates should be approached from a time dynamic perspective and address specific gender and age categories [14,17,18]. The best way to describe trends in health indicators is to evaluate the outcomes of past health policies and to ascertain the current health status of the population so that future improvements may be undertaken [19]. Currently, there is no research in South Africa, and to the best of our knowledge in sub-Saharan Africa, providing a dynamic image of a country’s burden of disease status from a spatial-temporal point of view. Most age and gender all-cause mortality risks are analyzed independently, either descriptively [7] or using disease mapping models [14,16,20,21]). However, several cause-specific mortality risks from certain diseases are age- and gender-dependent, for example, infectious-related deaths are more prevalent among young adults and children, and mortality from non-communicable disease are higher in the elderly [22]. Thus, independent analyses of age and gender mortality risks would not capture common and uncommon risk factor relationships between a number of age-gender mortality rates [16]. It becomes necessary to isolate common and age-gender-specific geographical patterns of mortality in a single model, which can be achieved by using the recently developed multivariate disease mapping models for multiple disease outcomes [3,10,11,13,15,16,23]. These models will enable us to assess similarities as well as differences between geographical risk patterns among age-gender groups purportedly sharing common risk profiles. These common factors may be thought of as proxies for unobserved health determinants shared by the multiple age and gender mortality risks. In the context of populations with limited resources, these methods are appealing due to the limitations of the available factors and disease data over the years. Additionally, by combining data from different age and gender groups, improvement in precisions and efficiencies of estimates, especially for rare mortality groups and causes, are obtained. 

This study is set in the sub-Saharan Africa region, which is in the midst of considerable epidemiological and demographic changes that are significantly affecting the region’s disease and mortality profiles [1]. These include the Human immunodeficiency virus infection/acquired immune deficiency syndrome (HIV/AIDS) epidemic, which has infected more than 30 percent of adults, and has resulted in a sizeable impact that has changed trends in many other diseases including tuberculosis (TB). There has also been a rise in lifestyle-related factors for non-communicable diseases (NCDs) which now account for a large proportion of adult deaths in the region. In South Africa, young adult mortality is increasingly more related to the impact of HIV/AIDS, TB, non-natural injuries, and emerging non-communicable diseases. We are not aware of any previous space and time dynamics analysis of mortality in South Africa based on the Mortality Statistics vital registration data, a very rich mortality data set spanning over a long period. Groenewald et al. [22] made the first attempt to assess and compare the cause of death profiles for each of the 52 health districts in South Africa. It is within these contexts of scarcity of such research methods and applications that this paper aims to explore geographical and temporal structures for several age and gender mortality risks jointly. In order to uncover the possibilities of unobserved ecologically distributed health services, disease burden, environmental influences and demographic factors responsible for the observed inter age-gender mortality correlations in South Africa, a shared spatial-temporal model with common and specific components is employed. These hidden factors may be reflecting the prevailing and changing distributions of age and gender mortality burden associated with changing epidemiological and demographic transitions over space and time in South Africa.

## 2. Statistical Models and Data Analyses

To study spatial-temporal trends of age and gender specific all-cause mortality from 1997 to 2013 in South Africa, a hierarchical Bayesian shared component spatial-temporal model [10] was undertaken using the municipality as the spatial unit for analysis. Here, $Y_{ijkt}$ is the number of all-cause deaths in municipality i during year t for gender j and age group k in South Africa, and $n_{ijkt}$ is the corresponding exposed population count. Several studies including Ocaña-Riola and Mayoral-Cortés [14], Schootman et al. [24], Richardson et al. [3], Tzala and Best [11] and Ocaña-Riola et al. [17] have used Bayesian hierarchical spatiotemporal methods to smooth observed small-area mortality rates.

The observed death counts $Y_{ijkt}$ are commonly modelled as conditionally-independent Poisson random variables with means $\mu_{ijkt}$ in a generalized linear model using a log link function. The means are modelled either as $\mu_{ijt}=n_{ijt}SDR_{ijt}$ or $\;\mu_{ijt}=\left(n_{ijt}R_{jt}\right)RR_{ijt}$, where $SDR_{ijt}$ and $RR_{ijt}$ are the age and gender specific mortality rate and mortality rate ratio at year t for gender j and age group k in municipality i.

(1) $$\log\left(\mu_{ijkt}\right)=\log\left(E_{ijkt}\right)+\log\left(RR_{ijkt}\right)$$

where $E_{ijkt}$ is the expected count calculated on the basis of the average age-gender-specific mortality rate over the entire observation period. The model (1) estimates the specific rate ratios of each municipality compared to the specific rate ratios of the whole country. In the modelling, we included main shared spatial, temporal, and space-time interaction, and gender-specific effects within the framework of the Besag, York, and Mollié [9] model. For this study, all spatially structured random effects were modelled using intrinsic conditionally autoregressive (ICAR) normal prior distribution to capture local dependence in space. For temporal trends, it has been observed that quadratic time effect captures most mortality trends [14,17]. However, to capture local dependence in time, the temporal terms were given first order random walk prior, which is simply a one-dimensional version of the ICAR normal prior distribution. The heterogeneity random effects terms were assumed to be distributed as multivariate normal prior distribution. In the modelling of spatial terms, there were considerations of the number of spatial fields to include, as these could fields be confounded and hence increase the uncertainty in the model [25]. 

Following Richardson et al. [3] and Manda et al. [13], a Bayesian spatiotemporal smoothing analysis is used where we use one shared “mortality and morbidity” spatial component common for males and females in each of the three age groups: a common space-time order 2 interaction for all the six age-gender groups. One female differential from the shared spatial pattern in each age group was included as well as age-gender specific heterogeneous effects, capturing possible extra-Poisson variation that is unexplained by the included random terms. We may take the specific component for age groups 15–64 as a proxy for infectious etiology in communicable diseases and unnatural deaths while taking the component for age groups 65 years and over as a proxy for non-communicable diseases [22]. Temporal random components were similarly modelled using shared time trend and female differential from the shared time trend within each age group.

Various parameters of the model were estimated using the WINBUGS Bayesian methods statistical software by running three parallel Gibbs sampler chains for 20,000 iterations from independent starting positions. Using a combination of trace plots and formal convergence diagnosis tools, 5000 iterations were deemed sufficient for convergence. The resulting combined sample of the remaining 45,000 iterations was used for posterior summaries.

## 3. Data Sources and Variables

In South Africa, death notification forms are medically certified and registered with the Department of Home Affairs (DHA). Statistics South Africa compiles and processes the death records forms into annual mortality reports [5,6,26] (Even though there are various sources of mortality data in South Africa [27], the vital death registration data are the best and most reliable source for mortality data in the country, and display very high levels of completeness [22]. We analyzed the mortality data for the period 1997 to 2013 and considered all-cause mortality using six age-gender age groups as follows: (0–14, 15–64, and 65 and over years) and gender (male and female). Age-gender population totals at the municipality administrative division from Statistics South Africa are only available for the national population and housing census years 1996, 2001, and 2011. For the non-census years between 1997 and 2011, we used an exponential growth rate to interpolate the specific age-gender municipality population totals using the formula: $P_{t+q}=P_{t}e^{rq}$ where $P_{t+q}$ is the ending population at q years later;$\;P_{t}$ is the beginning population, usually measured at census year t and r is the growth rate. However, for the years 2012 and 2013, extrapolation techniques were used to estimate the respective population totals based on the growth rate between 2001 and 2011. In line with the official publications, the number of death statistics used in this paper excludes stillbirths [5,28]. The specific rates of all causes of death in South Africa for each gender, age group, and year were then calculated.

Given the differential determinants and profiles of mortality risks across age groups in the country, expected death counts were derived for males and females separately in each age group using  R^j(k)=∑i,tYij(k)t∑i,tnij(k)t the average countrywide death rate for gender j(1=male,2=female) in age group k (k=1 (0–14 years), 2 (15–64 years), 3 (65 years or over)) over the entire observation period 1997–2013. In the absence of covariate effects, $n_{ijkt}\;{\hat{R}}_{j\left(k\right)}$ is just the expected number of deaths in each gender j at time  t, as denoted by $\;E_{ijt}$. The estimated baseline rate defined above is known as internal standardization, and the ratio of observed to expected deaths  YijktEijkt is termed the standardized mortality ratio (SMR), which is just the estimate of $RR_{ijkt}$ under maximum likelihood methods. In this study, an age and gender specific SMR of one was taken to indicate that the observed respective death count is equal to that of the expected count. Rather than using the actual SMR, a probability that SMR was greater than one was computed and a probability value of 0.8 or above would mean high mortality risk. On the other hand, if this probability is below 0.2 then the respective area is of low risk [13]. 

Thus, the mortality data we considered here are recorded for J=2 genders (male and female) and K=3 age groups (0–14, 15–64, 65 and over years) for each of the I=234 municipalities over an observation period of T=17 years (1997–2013), yielding a total of 23,868 observations. This number of observation years is adequate to assess our interest in the temporal component to explore changes in rates over a relatively long period. Furthermore, epidemiological and demographic issues are of interest because of possible variations in disease and environmental exposures for various population subgroups. In addition, the nutritional, epidemiological, and demographic profile of the municipalities would most likely have evolved over the period of interest.

## 4. Results

### Observed Numbers and Rates

Table 1 shows the distribution of all-cause deaths by gender and age groups across all municipalities in 1997, 2005 and 2013. The annual number of notified and registered deaths increased from just over 300,000 in 1997 to about 624,782 in 2006, then decreased down to just over 458,933 in 2013. The annual number of deaths rose by a massive 93% between 1997 and 2006, and then decreased by 26.5% between 2006 and 2013. The increase may be due to population growth and better reporting coverage. The average observed number of deaths for males for the age group 0–14 was 154 (range of 0–3448) per municipality per year, while for the age groups 15–64 and 65+ years, the numbers were 723 (3–15,470) and 249 (1–4964), respectively, per municipality per year. For females, the average numbers of observed deaths for the age groups 0–14, 15–64, and 65+ years were 133 (0–2860), 592 (2–13,456), and 318 (1–5894 range), respectively, per municipality per year. This shows variations in deaths recorded across the municipalities in the country, which may be indicative of variation in the quality and completeness of death reporting [22]. The distribution of the number of deaths for each of the municipalities for each age-gender grouping in three selected years, 1997, 2005, and 2013, was investigated and the results showed high variability in all age and gender groups.

**Figure 1:**
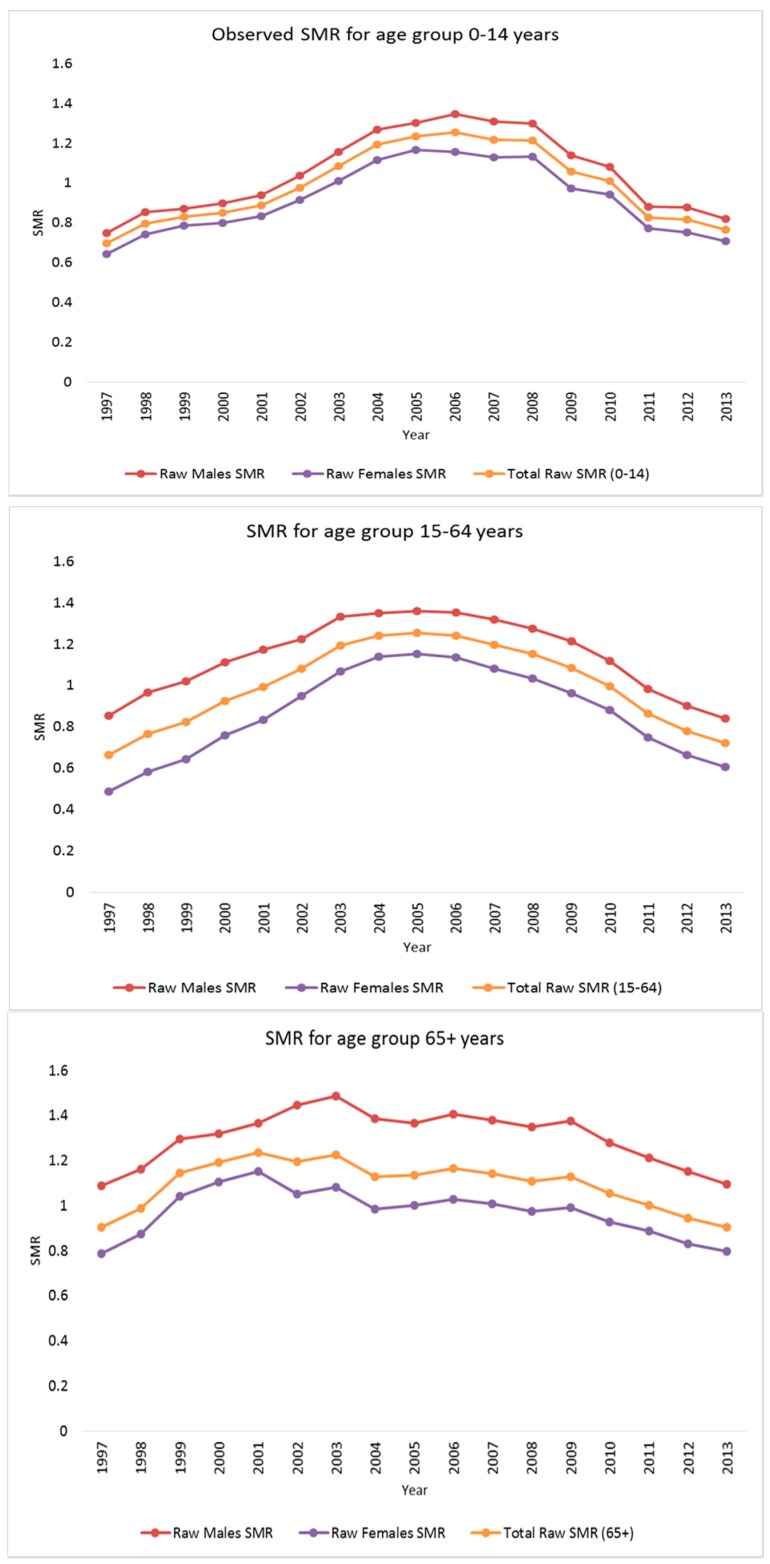

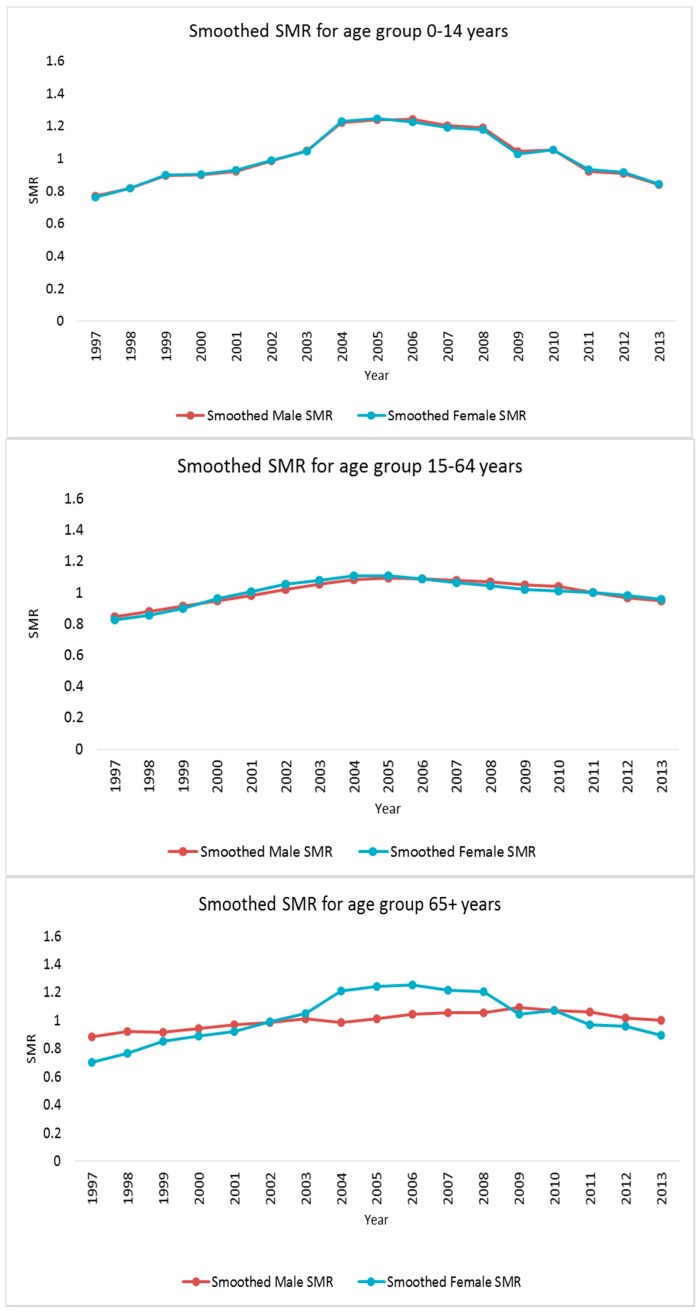
Overall time series plots of the raw and posterior median smoothed standardized mortality rate ratios (SMRs) for males and females within each of the three age bands.

Specific mortality rates were calculated as the number of deaths per 10,000 residents in South Africa for each year, gender, and age group. For males in the age group 0–14 years, the average mortality rate was 49.5 (range: 0–274.5) per municipality per year and for the 15–64 and 65+ years age groups, the mortality rates were 127.4 (4.8–942.3) and 518.1 (18.4–4528.1), respectively. For females, the average mortality rate per municipality per year for age groups, 0–14, 15–64, and 65+ were 40.1 (0.0–242.5), 98.1 (4.2–927.6), and 592.3 (31.9–5699.0), respectively. Figure 1 displays the time trends of the SMRs by gender within each age group over the seventeen years studied. Overall SMRs are higher for all age-gender groups around 2002–2008 (with the highest SMRs around the 2005–2006 period) and decrease afterwards, indicating that the time trends are going in the same direction.

**Figure 2:**
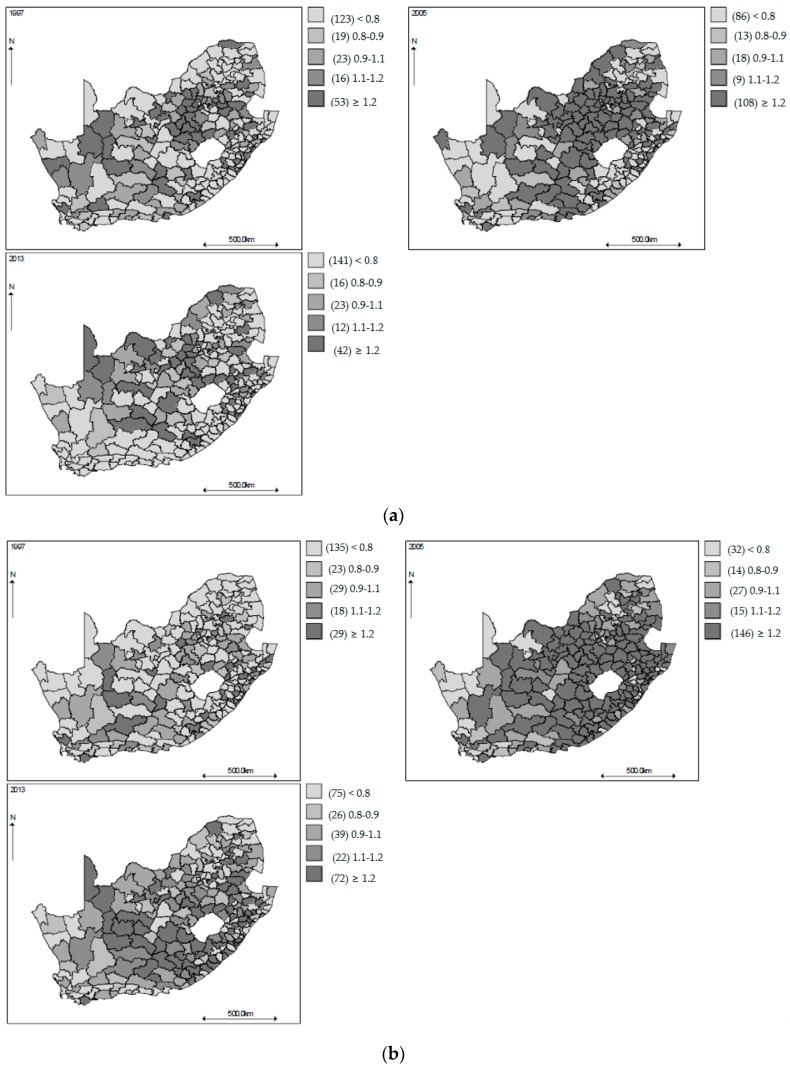

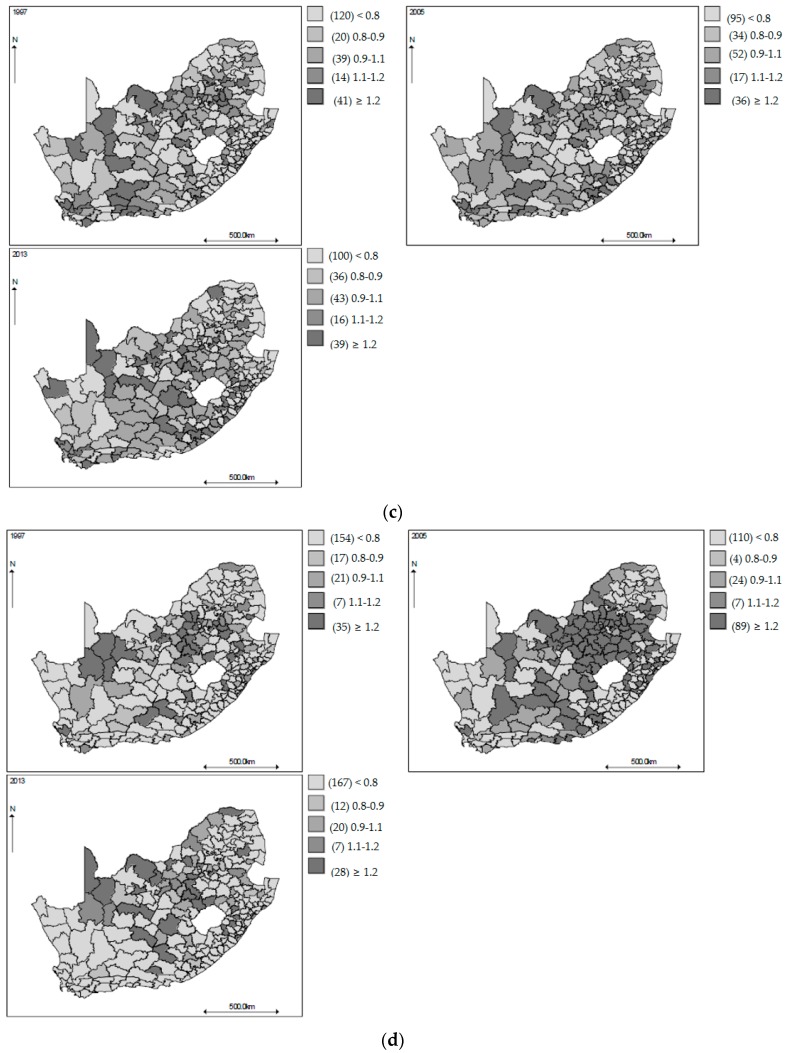

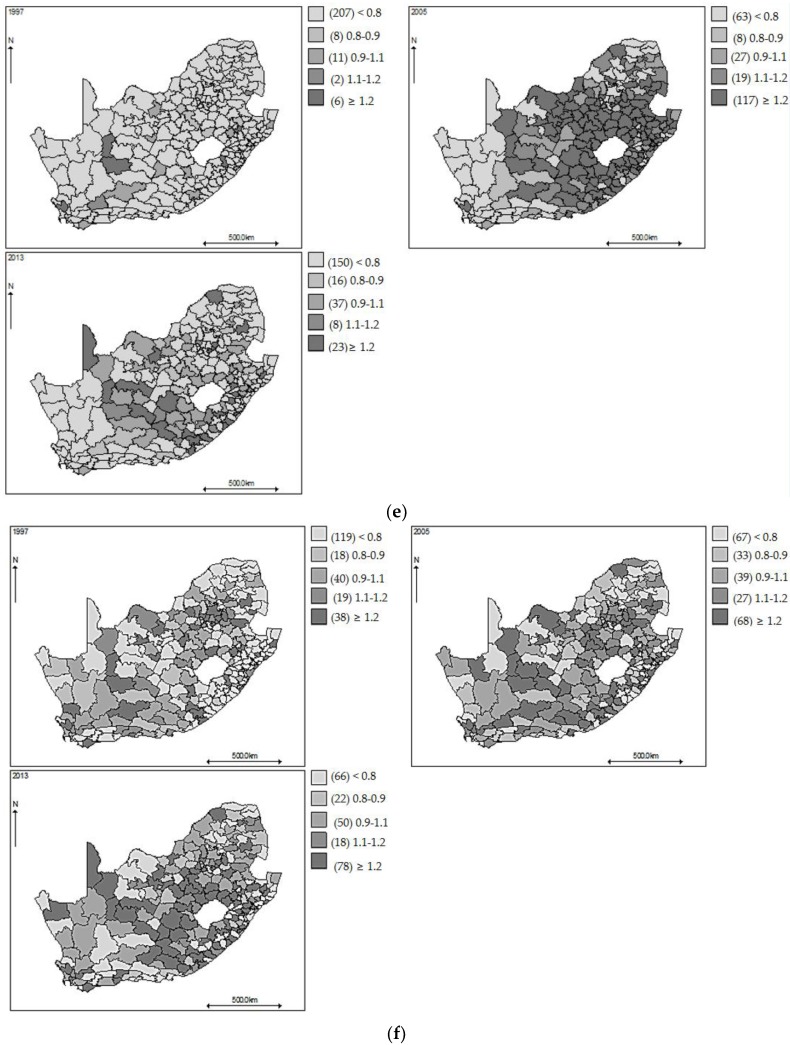
(**a**) Maps of municipality all-cause raw standardized mortality ratios for men aged 0–14 years in 1997, 2005, and 2013, South Africa; (**b**) Maps of municipality all-cause raw standardized mortality ratios for men aged 15–64 years in 1997, 2005, and 2013, South Africa; (**c**) Maps of municipality all-cause raw standardized mortality ratios for men aged 65 years or over in 1997, 2005, and 2013, South Africa; (**d**) Maps of municipality all-cause raw standardized mortality ratios for female aged 0–14 years in 1997, 2005, and 2013, South Africa; (**e**) Maps of municipality all-cause raw standardized mortality ratios for female aged 15–64 years in 1997, 2005, and 2013, South Africa; (**f**) Maps of municipality all-cause raw standardized mortality ratios for female aged 65 years or over in 1997, 2005, and 2013, South Africa.

Figure 2 displays the geographical patterns of the observed SMRs in 1997, 2005, and 2013. Overall higher SMR are observed for male mortality in the 0–14 and 15–64 years age groups (Figure 2a,b) in 2005 around the eastern and south-eastern and north central regions, respectively, whereas the pattern for males aged 65 years and over is less clear. For the females, higher rates are also observed in the 0–14 years age group (Figure 2d) in 2005 in the middle north-eastern region of South Africa, but for females in the 15–64 years age group (Figure 2e), higher SMRs in the year 2013 are observed mainly in the north-western and south-eastern regions, indicating a shift in time trends from the males in the same age group. As time progresses, females aged 65 years and over, have lower SMRs, indicating that this group’s time trends are going in the opposite direction to most age-gender groups studied here. It is evident that there is a large amount of noise in the displayed maps, which makes it difficult to discern a clear pattern, and modelling would be necessary to extract the steady geographies.

**Figure 3:**
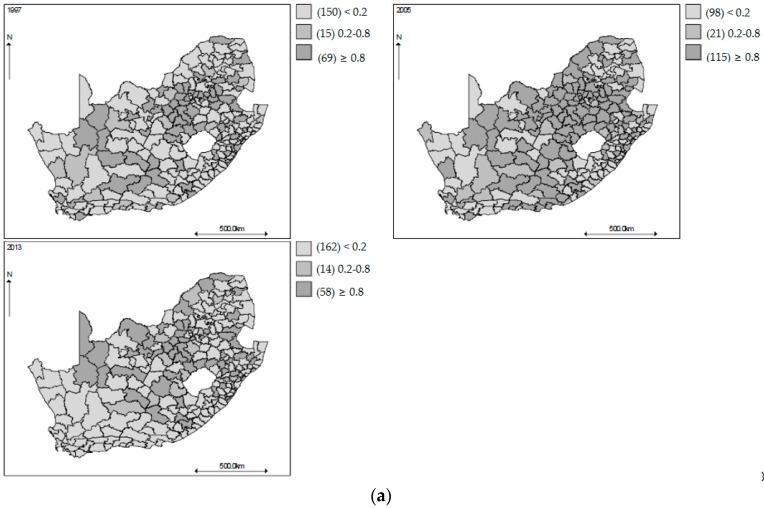

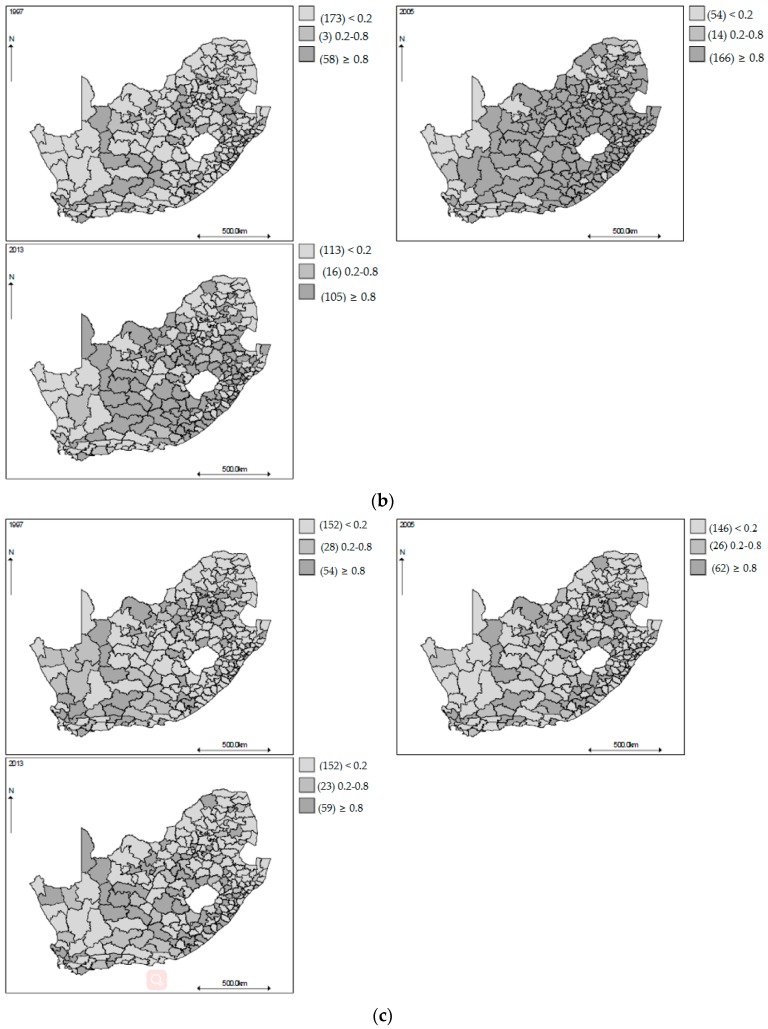

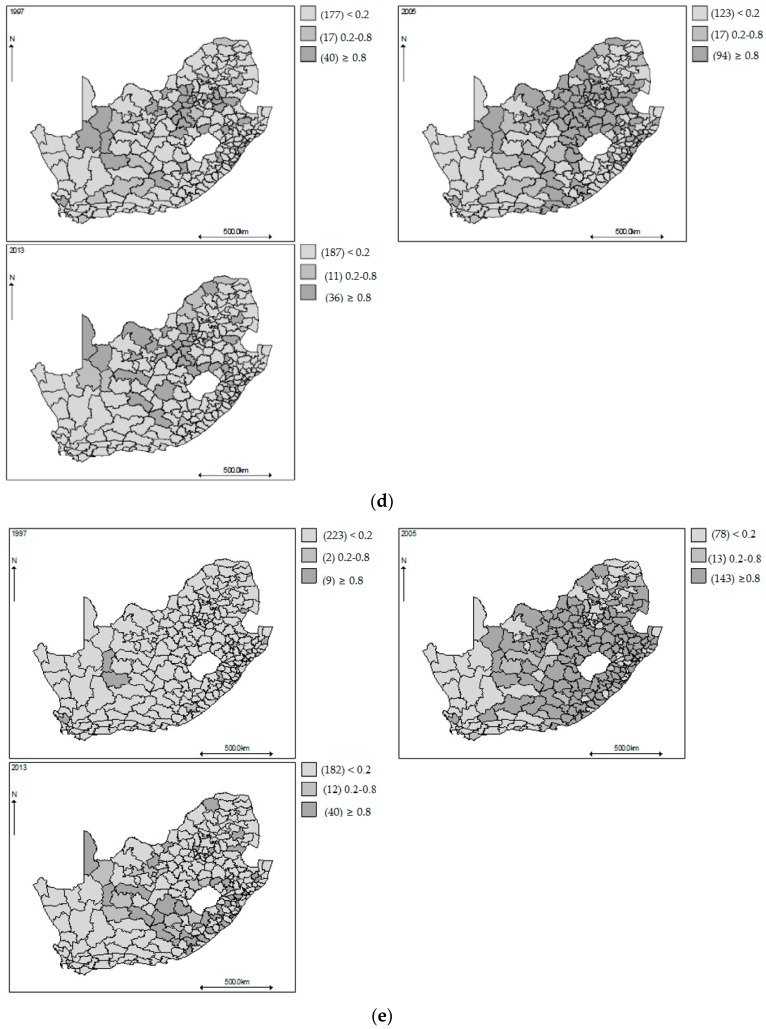

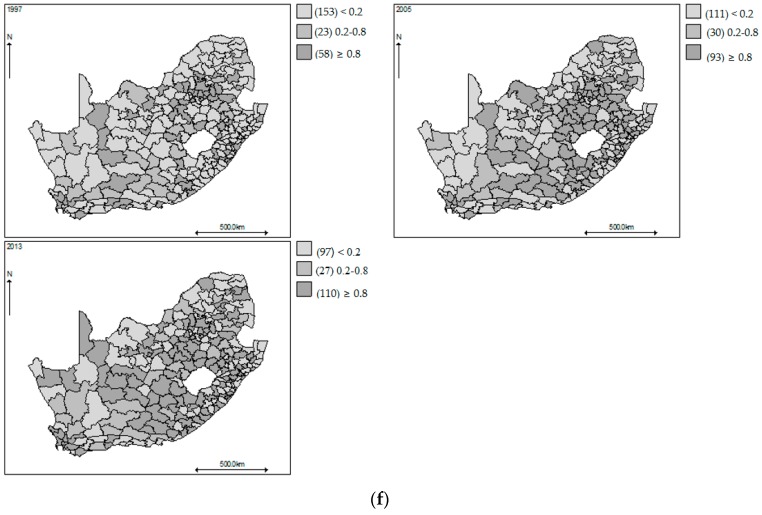
(**a**) Mapped posterior probability that the all-cause smoothed standardized mortality ratios for males aged 0–14 years exceed 1 in 1997, 2005, and 2013, South Africa; (**b**) Mapped posterior probability that the all-cause smoothed standardized mortality ratios for males aged 15–64 years exceed 1 in 1997, 2005, and 2013, South Africa; (**c**) Mapped posterior probability that the all-cause smoothed standardized mortality ratios for males aged 65 years or over exceed 1 in 1997, 2005, and 2013, South Africa; (**d**) Mapped posterior probability that the all-cause smoothed standardized mortality ratios for females aged 0–14 years exceed 1 in 1997, 2005, and 2013, South Africa; (**e**) Mapped posterior probability that the all-cause smoothed standardized mortality ratios for females aged 15–64 years exceed 1 in 1997, 2005, and 2013, South Africa; (**f**) Mapped posterior probability that the all-cause smoothed standardized mortality ratios for females aged 65 years or over exceed 1 in 1997, 2005, and 2013, South Africa.

**Figure 4:**
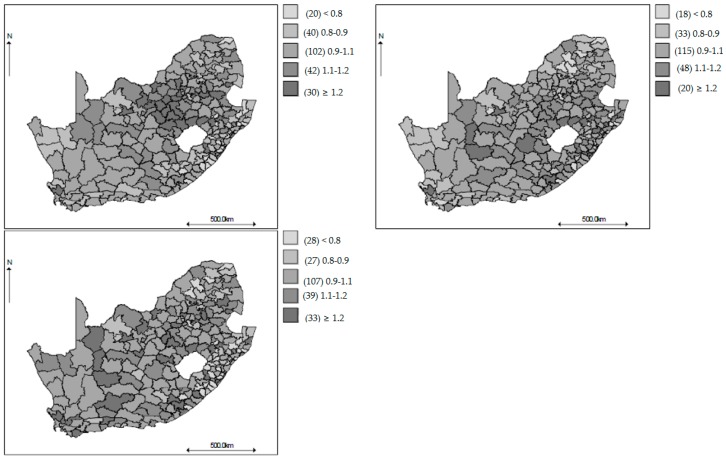
Maps of the posterior median female and male aged 0–14 years (**Top left**), female and male aged 15–64 years (**Top right**), and female and male aged 65 years and over (**Bottom left**) shared components.

Figure 3 shows geographical variations of the age-gender specific smoothed standardized mortality ratios in the municipalities of South Africa, in 1997, 2005 and 2013. The values plotted are the probability that the age-gender specific mortality rates in each municipality is greater than 1 using the respective countrywide mortality rate as the reference. The municipalities with probability greater than 0.8 and with probability less than 0.2 are taken to have experienced the age-gender specific all-cause mortality rate significantly greater and lower than the respective country-wide age-gender specific mortality rate, respectively. During the period covering the decade of 2000, there was a group of municipalities with age-gender specific all-cause mortality rates significantly different from the respective age-gender specific all-cause national rates. The patterns are similar to those depicted in the maps of observed SMRs, but now they are more discernable, showingevidence of spatial heterogeneity with higher SMRs in the southern-eastern belts mainly for the 15–64 age groups in 2005. At the start and end of the study period, the relative risk (RR) maps show that most of the municipalities have lower mortality risk across most of the age-gender groups. The same patterns are observed from the time plots of the smoothed SMRs over the entire period (Figure 1). The gender shared components are presented in Figure 4 for each age group, where the pattern of higher SMRs around the eastern regions of SA is observed for both the 0–14 and 15–64 age groups, but less contrast in the SMRs for the 65 years and older age group.

The analyses and results presented are using a municipality administrative division in order to provide evidence at the local government level of planning. We are aware that a different grouping unit could have resulted in different spatial patterns and interpretations. This is a problem of modifiable areal unit problem (MAUP) in spatial analyses where an analysis based on particular grouping unit may accidently misrepresent or overstate the actual mortality risk variations [29]. Even if the data are grouped at the same level for analysis, the way the grouping scheme is used for spatial analysis may accidentally lead to misinterpretation of the spatial patterns. Changing boundaries of municipalities to assess changes to the overall spatial patterns in the estimated age-gender all-cause mortality rates could have been a possible option, but this was beyond the scope of the paper. However, we performed additional analyses by choosing a coarser scale at the provincial administrative division. The general spatial patterns were slightly changed, but not to an extent that affected the general substantive conclusions.

## 5. Conclusions

This study has undertaken a novel approach using dynamic spatial-temporal distributions to provide a better understanding of the evolvement of mortality risks over time and space in South Africa. As commented in Ocaña-Riola and Mayoral-Cortés [14] and Ocaña-Riola et al. [17], such an analysis will contribute towards a reflection on the past, present, and future of mortality in South Africa. The adopted modelling approach has allowed for common and age-gender specific components of mortality effects to be identified [3,13]. We found differences in mortality rates from 1997 to 2013, with high rates in the period around 2005, especially for the 15–64 years age group for both males and females. There were also differences in mortality rates between municipalities, which may in part reflect differences in health care quality and age distribution and the prevailing major burden of diseases. 

South Africa is undergoing demographic, epidemiological, and nutritional changes that have influenced lifestyle-related morbidity and mortality. Furthermore, the 15–64 years age group is very crucial in studying the effect of HIV/AIDS and TB related mortality in addition to mortality related to NCDs. Thus, the results presented in this study will provide policymakers with empirical evidence that will help them to map policies aimed at narrowing the disparity in health care services for municipalities. Some of these initiatives are already being attended to, as detailed within the National Department of Health Strategic Plan 2014/15–2018/19 [30]. These initiatives include reducing the burden of HIV/AIDS, TB, and non-communicable diseases, increased primary health access to families and communities, and achieving universal health care coverage.

Possible limitations of our study include under-reporting and the completion of the mortality data, which may affect and confound the observed rates and their interpretation [22]. Furthermore, the quality of the data has been found to vary sub-nationally in South Africa [22]. The use of interpolated population totals using the three sets of census population data from 1996, 2001, and 2011 could have resulted in inaccuracies of the mid-year population totals across the six age-gender groups for the entire 17 years. This may have affected the calculations of some of the mortality rates, especially for small and sparser municipalities. The analysis did not account for important risk factors (for example, health service availability and disease burden), environmental influences, or demographic factors. These were unavailable because they are difficult to measure or obtain. However, a benefit of employing the multivariate spatial-temporal model is its ability to account for such unmeasured exposures that may be common among the diseases considered. Mortality was analyzed in very course age intervals; this may mask differentials in mortality levels. Using finer age intervals could ensure that age differences between municipalities are removed. Unlike in developed countries, the population data that was made available from Statistics South Africa, although considered reliable, only had detailed population data on the age-bands analyzed (0–14, 15–64, and 65+), even so, only for census years 1996, 2001, and 2011. Most mid-year population estimates in South Africa are at the regional level, and not often given by age and gender.

We are also aware that several multivariate mapping models have been proposed, and any could have been applied to our multiple mortality outcomes studied here. We are in the process of undertaking model validation to identify the best predictive model from a number of candidate multivariate spatial models. Furthermore, there are several mortality data sources in South Africa that could have been used for the analyses contained here [27]. Such a wealth of data may permit mortality data triangulation by, for example, combining different data sources for mortality to estimate sub-national relative risk profiles in adult mortality, as suggested by Dorrington and Timæus [31]. We are developing Bayesian multivariate models to perform data source triangulation using vital registration, census data, and in-hospital mortality records for the year 2011. Of note, the vital death statistics data have been found to be of high coverage, especially at the level needed for the mapping in this study. Another limitation would be the use of the 15–64 age group in the models. This could be split into two separate age groups, such as 15–44 and 45–64, to ensure that age differences between municipalities are removed. However, the unavailability of data at that age band disaggregation by gender would limit such make an undertaking.

In conclusion, this study has shown the utility of using recently developed shared component spatiotemporal models to fit a dynamic geographical distribution of mortality for different gender and age groups simultaneously. This approach has allowed for the identification of similar and divergent patterns in geographical and temporal distributions of age and gender mortality risks in South Africa in a multivariate way. This may provide more convincing evidence of periods and areas at greatest risk for mortality and therefore will contribute towards a reflection on the past, present, and future trends in age-gender specific all-cause mortality in different areas in order to help enable better local policy planning.

## Figures and Tables

**Table 1:** Distribution of deaths by gender and age groups per municipality in South Africa, 1997, 2005, and 2013.

Male Mortality															
	0–14-Year-Old Age Group					15–64-Year-Old Age Group					≥65-Year-Old Age Group				
Year	Deaths	Min	Max	Mean	SD	Deaths	Min	Max	Mean	SD	Deaths	Min	Max	Mean	SD
1997	23,492	1	2260	100.39	229.47	105,734	6	9196	451.85	1107.6	47,549	1	3532	203.2	450.89
2005	45,153	1	3253	192.96	400.97	202,348	14	14,921	864.74	1782.4	58,628	3	4352	250.54	524.15
2013	28,477	1	1883	121.7	231.07	147,272	13	9930	629.37	1262.87	60,769	3	4964	259.7	531.85
**Female Mortality**															
	**0–14-Year-Old Age Group**					**15–64-Year-Old Age Group**									
Year	Deaths	Min	Max	Mean	SD	Deaths	Min	Max	Mean	SD	Deaths	Min	Max	Mean	SD
1997	20,304	0	2004	86.77	200.23	65,577	2	5477	280.24	638.29	53,883	3	4107	230.27	532.54
2005	40,040	2	2791	171.11	353.57	184,237	8	13,279	787.34	1495.73	76,153	4	5528	325.44	689.74
2013	24,388	0	1665	104.22	202.7	110,345	2	7274	471.56	903.68	80,182	3	5894	342.66	679.57
